# Examining Tweet Content and Engagement of Canadian Public Health Agencies and Decision Makers During COVID-19: Mixed Methods Analysis

**DOI:** 10.2196/24883

**Published:** 2021-03-11

**Authors:** Catherine E Slavik, Charlotte Buttle, Shelby L Sturrock, J Connor Darlington, Niko Yiannakoulias

**Affiliations:** 1 School of Earth, Environment and Society McMaster University Hamilton, ON Canada; 2 Division of Epidemiology Dalla Lana School of Public Health University of Toronto Toronto, ON Canada; 3 School of Geography and Environmental Management University of Waterloo Waterloo, ON Canada

**Keywords:** COVID-19, coronavirus, pandemic, public health, Twitter, social media, engagement, risk communication, infodemiology, content analysis

## Abstract

**Background:**

Effective communication during a health crisis can ease public concerns and promote the adoption of important risk-mitigating behaviors. Public health agencies and leaders have served as the primary communicators of information related to COVID-19, and a key part of their public outreach has taken place on social media platforms.

**Objective:**

This study examined the content and engagement of COVID-19 tweets authored by Canadian public health agencies and decision makers. We propose ways for public health accounts to adjust their tweeting practices during public health crises to improve risk communication and maximize engagement.

**Methods:**

We retrieved data from tweets by Canadian public health agencies and decision makers from January 1, 2020, to June 30, 2020. The Twitter accounts were categorized as belonging to either a public health agency, regional or local health department, provincial health authority, medical health officer, or minister of health. We analyzed trends in COVID-19 tweet engagement and conducted a content analysis on a stratified random sample of 485 tweets to examine the message functions and risk communication strategies used by each account type.

**Results:**

We analyzed 32,737 tweets authored by 118 Canadian public health Twitter accounts, of which 6982 tweets were related to COVID-19. Medical health officers authored the largest percentage of COVID-19–related tweets (n=1337, 35%) relative to their total number of tweets and averaged the highest number of retweets per COVID-19 tweet (112 retweets per tweet). Public health agencies had the highest frequency of daily tweets about COVID-19 throughout the study period. Compared to tweets containing media and user mentions, hashtags and URLs were used in tweets more frequently by all account types, appearing in 69% (n=4798 tweets) and 68% (n=4781 tweets) of COVID-19–related tweets, respectively. Tweets containing hashtags also received the highest average retweets (47 retweets per tweet). Our content analysis revealed that of the three tweet message functions analyzed (information, action, community), tweets providing information were the most commonly used across most account types, constituting 39% (n=181) of all tweets; however, tweets promoting actions from users received higher than average retweets (55 retweets per tweet). When examining tweets that received one or more retweet (n=359), the difference between mean retweets across the message functions was statistically significant (*P*<.001). The risk communication strategies that we examined were not widely used by any account type, appearing in only 262 out of 485 tweets. However, when these strategies were used, these tweets received more retweets compared to tweets that did not use any risk communication strategies (*P*<.001) (61 retweets versus 13 retweets on average).

**Conclusions:**

Public health agencies and decision makers should examine what messaging best meets the needs of their Twitter audiences to maximize sharing of their communications. Public health accounts that do not currently employ risk communication strategies in their tweets may be missing an important opportunity to engage with users about the mitigation of health risks related to COVID-19.

## Introduction

### Background

On January 25, 2020, the first case of COVID-19 was reported in Canada in a man who had recently traveled to Wuhan, China, where the virus was first identified [1]. By mid-March, in the days after the World Health Organization (WHO) declared COVID-19 to be a pandemic, Canadian public health officials began to issue warnings against all nonessential travel, and soon local community transmission was confirmed to be the primary source of the transmission of cases in the country. By March 22, all Canadian provinces entered a state of emergency, ordered all nonessential businesses to close, and restricted public gatherings [2].

During this time, public health agencies and officials emerged as the de facto leaders and primary decision makers for setting evidence-based public health policies, practices, and norms. Daily updates from medical officers of health and other public health experts would set the course for how each jurisdiction would respond to COVID-19 and outline the public’s role in “flattening the curve.” Some early research suggests that Canadians listened to these messages and followed public health recommendations [3], and also stayed home, particularly in the early weeks of the pandemic, as demonstrated by decreases in the levels of people’s movements tracked through Google’s Community Mobility Reports [4].

### Public Health Administration in Canada

The roles and responsibilities of Canadian public health institutions and individuals differ across the country and by levels of government. As a result, it is not always clear where the public should go to access and retrieve information during a health crisis. For example, the federal government’s main role is to communicate national case numbers to all Canadians, coordinate control measures across provinces, and provide updates on national issues such as travel and the delivery of medical supplies. These activities are undertaken mostly by the Public Health Agency of Canada (PHAC), which was established as a separate agency of the federal department of health specifically to improve responses to infectious disease outbreaks after the severe acute respiratory syndrome (SARS) outbreak of 2003 [5].

Conversely, Canadian provinces are responsible for leading the emergency response, whereby ministries of health are tasked with communicating provincial updates on case counts, conducting surveillance and monitoring, providing guidance on infectious disease control measures and policies, and testing and screening practices [6]. Some provinces also operate regional and/or local-level public health units, which communicate information about local public health measures they have set and enforced based on provincial emergency orders. In addition to provincial health ministries, Ontario, British Columbia, and Quebec also have separate provincial-level public health agencies to provide scientific and technical advice on public health matters, conduct specialized data analytics, and provide updates on provincial testing capacity or other expert advice related to infectious disease control. Each of the levels of government described (federal, provincial, and regional/local where they exist) also appoints a medical officer of health to lead public health efforts in their respective jurisdiction, who often holds degrees and training in both medicine and public health.

The diversity in public health responses and responsibilities between institutions and individuals led virtually every Canadian province to take a different approach to crisis communications and information dissemination related to COVID-19. For example, provinces such as British Columbia have put their provincial medical officers of health in the spotlight, while others, including Ontario, have opted to have elected officials such as local premiers or health ministers lead some of the response. Occasionally this has resulted in contradictory messaging from multiple spokespeople, leaving the public confused and unsure whose guidance to follow [7]. This issue is particularly relevant on social media platforms like Twitter, where an abundance of information and misinformation has resulted in an infodemic [8], which can produce uncertainty and anxiety for individuals navigating an information overload [9]. Inconsistencies in health messaging can also erode public trust in the competence and credibility of public health agencies and decision makers [10].

### Role of Public Health Agencies on Twitter

One of the ways in which the public has stayed informed on key information and updates on COVID-19 has been through the use of social media applications. Twitter in particular reported its biggest ever annual gain in daily users globally during the pandemic, which was up by 24% year over year during the first 3 months of 2020 [11]. On the one hand, an increase in Twitter users can lead to a more informed public as past research has suggested that a high proportion of users have identified Twitter as a major source of news for them [12]. On the other hand, an increase in Twitter users may also increase exposure to incorrect information or outright misinformation about COVID-19 [13]. Although the mass media have historically played a major role in the flow of information between public officials and the public during crises, the increased use of social media applications like Twitter has allowed members of the public to connect with governmental organizations and individuals more directly, largely circumventing the need to follow other unofficial communicators [14].

During a health crisis such as a pandemic, the role of public health agencies and officials as communicators of timely and accurate information is especially crucial in helping the public form accurate perceptions of health risks and adapt their behaviors in ways that are necessary to mitigate risks [15]. In fact, social media platforms like Twitter have enabled users to seek and share information and news updates during past crises to help reduce feelings of uncertainty and cope with threats [16]. Some early studies of the COVID-19 pandemic have highlighted the need for public health officials to utilize more communication channels and exert their influence as risk communicators in a time when the global need for expert information and advice has peaked [17]. Information posted to social media especially at the early stages of any crisis or risk event tends to garner more traction online as users seek out updates. As such, it is critical for risk communicators to establish an early online presence and engage those users from the beginning [18].

One way to assess the online influence of Twitter accounts is to examine the engagement that their tweets receive. This can indicate how much an account’s communications are being seen, studied, and shared. Tweet engagement can be indicated by various measures including the number of retweets (ie, shares of a tweet), likes (ie, number of times a user has seen and acknowledged or agreed with a tweet), or replies (ie, number of times a tweet has been commented on or responded to). Retweets in particular have been identified as an effective measure of engagement as they can indicate both the level of user agreement with a message and also the level of diffusion that message has undergone based on how many shares it has amassed from the original tweeter [19]. Beyond providing confirmation that some information or message has been disseminated to the public, quantifying tweet engagement based on retweets can provide a direct measure of the impact of that tweet on users. Some research has suggested that source credibility plays a role in garnering engagement; health agencies or individuals who appear to provide trustworthy information may be able to leverage their perceived legitimacy to gain more retweets and disseminate their information more broadly [20]. Researchers have also identified engagement strategies that can be used to increase user engagement to tweets. These strategies include the use of hashtags, URLs, user mentions (ie, direct mention of other Twitter user accounts), and media (eg, images or videos) [21].

### Prior Work

Our study builds on past research that has examined the use of Twitter specifically by public health departments, agencies, and organizations. Most studies tend to focus on either examining the relationship between tweet features and levels of engagement and/or analyzing the content of the tweets to characterize the tweeting practices of particular accounts. For example, in a study of tweet engagement strategies used by 25 federal health agencies in the United States, it was found that hashtags, URLs, and user mentions were associated with an increased frequency of retweets [22]. In prior content analyses of tweets by state and/or local health departments in the United States, studies classifying the purpose of tweets (eg, whether tweets served to inform users or prompted them to perform some activity) have found that health departments mostly use Twitter to share health information [23-26]. However, other research has suggested that tweets whose function was to promote an action received more retweets than those with other functions [21]. Our work is also guided by research about prior pandemics such as tweeting trends during the H1N1 outbreak [27] and tweets covering Ebola health risks [28-30].

Previous work summarizing best practices in risk communication during broader risk events [31,32], as well as previous public health crises [33-35], underscore the importance of incorporating effective risk communication elements in messaging in order to reduce harm, clarify facts, and address public concerns. Beyond simply providing accurate descriptions of risks about the likelihood and consequences of harms, effective risk communication practices on Twitter may also include the use of messages promoting self-efficacy (ie, an individuals’ beliefs that they have the ability to take action), providing reassurances, acknowledging concerns and uncertainties surrounding the situation, and indicating coordination of actions between experts. These strategies are viewed as important tools for organizations to augment their credibility and diffuse public fears [36]. Some literature has also noted the importance of applying strategic risk messaging across different outbreak phases by first focusing on information accuracy, then moving to reassurances to reduce uncertainty, and lastly, by emphasizing self-efficacy through individual actions and preventive measures [37].

### Study Goal

The goal of our study was to characterize the content and level of engagement of COVID-19 tweets made by Canadian public health agencies and decision makers. Further, we propose recommendations for ways through which health agencies and decision makers could adjust their tweeting practices about COVID-19 and other future health crises to improve risk communication and maximize engagement. Our study seeks to answer four primary research questions (RQs):

- RQ1: which types of Canadian public health agencies and decision makers tweeted the most about COVID-19 and when?
- RQ2: how much engagement did tweets by Canadian public health agencies and decision makers receive during COVID-19? How did engagement change over time by account type?
- RQ3: did tweets containing Twitter engagement strategies receive more retweets than those that did not? How did the use of engagement strategies vary by account type?
- RQ4: did tweets from Canadian public health agencies and decision makers that employed a particular message function and risk communication strategy receive more retweets than others? How did the use of risk communication strategies in tweets from Canadian public health agencies and decision makers change over time?

## Methods

### Data Collection

A comprehensive list of Canadian public health institutions, agencies, and leaders was compiled after conducting a scoping review of provincial government websites. The resulting list of agencies and decision makers from this initial search was cross-referenced with the “Structural Profile of Public Health in Canada,” a resource published by the National Collaborating Centre for Healthy Public Policy [38] that summarizes how public health is organized federally, provincially, and regionally across Canada. The names of each of these agencies and individuals were then manually searched using the Twitter interface to narrow the list to include only those that had a Twitter account (n=128). This list of agencies and decision makers was then used to pull tweets for the identified key players in Canadian public health communication on Twitter.

Twitter data were downloaded using the Twitter API accessed through R using the *rtweet* package (The R Foundation) [39]. An R script was created to go through the list of 128 identified Twitter accounts and download the most recent 3200 tweets of each account, which reflects the maximum number of tweets allowed for account-specific searches as imposed by the application programming interface for Twitter. Data were originally collected on May 23 but were recollected every day from June 1 to July 10 using an automated script that iteratively updated the number of interactions with past tweets (such as likes or retweets) and collected new tweets published during the month of June. The Twitter data set contained tweet-level data including the author’s account name, Twitter handle, number of followers at the time of download; the date and time the tweet was published; whether the tweet was an original tweet or a retweet; the tweet’s text, hashtags, user mentions, URLs, favorite and retweet count; and whether the tweet contained media (eg, image).

The Twitter data collected between May 23 and July 10 yielded 303,428 tweets from February 2010 to July 10, 2020. The data were then narrowed down to only include tweets authored between January 1, 2020, and June 30, 2020, resulting in 71,014 tweets. This time period was selected as China first reported the outbreak of the novel coronavirus to the WHO on January 1, 2020. Of the 128 Twitter accounts we sampled, 118 had authored tweets between this period. Finally, retweeted tweets were excluded, resulting in 45,310 tweets.

Most tweets in the sample were single standalone tweets within the tweet character limit, but some comprised longer tweet threads. In these cases, tweets were turned into threads using the following procedure. First, all tweets were sorted by publisher and date and time. Next, all tweets that were identified as self-replies (based on the “reply_to_screen_name” variable) were treated as part of a tweet thread. The start of a tweet thread was the tweet that immediately preceded the chain of tweets that were self-replies. This starting tweet, and all subsequent self-replies, were combined into a tweet thread. These tweets and tweet threads (n=32,737) will be referred to simply as tweets throughout the remainder of this paper. The tweets were then classified by whether they were about COVID-19 or not, based on whether they contained any of the following keywords: “covid*,” “coronavirus,” “ncov,” “distanc*,” “pand*,” “tracing,” “testandtrace,” “curve,” “stayhome,” “handwashing,” “mask,” and “masque.” These keywords were identified by scanning tweets within the sample and noting commonly used words in French- and English-language tweets describing COVID-19. During this scan, it was found that most French-language tweets about COVID-19 used #COVID19 to denote the tweet’s topic relevant to COVID-19, which was a less common practice among English-language tweets. Hence, a larger number of English-language keywords were required to classify whether an English-language tweet was about COVID-19. This selection process resulted in 6982 tweets about COVID-19.

Additionally, we downloaded the publicly available COVID-19 data set from the Government of Canada [1] to plot active case counts over time alongside tweeting trends by public health accounts. The national COVID-19 data set aggregated case counts by the date that the case data were submitted to PHAC rather than the date that the cases were confirmed by the local health authority that collected the data.

### Data Analysis

To classify the public health accounts by type, each account was categorized as belonging to either an agency or a decision maker. If the Twitter account belonged to an agency, it was classified as being one of 3 types: public health agency (ie, a federal or provincial public health agency, distinct from a health ministry due to its focus on public health), a regional or local health department (ie, a public health department that offers public health programs or services to communities at a scale smaller than the province), or a provincial health authority (ie, provincial health ministries or health authorities). If the account belonged to a decision maker, it was classified as being one of 2 types: medical officer of health (ie, the chief medical health officer of Canada and of each province, and regional or local medical health officers responsible for public health in smaller communities) or provincial minister of health (ie, elected government official who oversees health and public health agencies).

Tweet engagement was measured using retweet count. For tweet threads that contained multiple tweets, the maximum number of retweets obtained for any single tweet in the thread was used to measure engagement (rather than the average or total number of retweets for all tweets in the thread) to avoid double-counting or high-biased engagement.

Next, accounts were classified based on province, where applicable, or were otherwise identified as a “national” account (eg, PHAC and Canada’s chief medical officer). This was done to select a stratified random sample of 501 tweets about COVID-19 for a qualitative content analysis (see Multimedia Appendix 1 for process flow chart). Since public health services and policies are primarily administered at the provincial level in Canada, we wanted our subset of tweets to capture enough geographic variation in the accounts across various provinces. We also wanted our subset of tweets to capture enough variation in tweeting by account type. Therefore, we used a stratified random sample with replacement using proportional weighting to randomly select tweets across various strata based on the number of tweets each stratum contributed to the total sample. These comprised of 8 regional strata, which included British Columbia, Alberta, the Prairies (Saskatchewan and Manitoba), Ontario, Quebec, the Maritimes (Nova Scotia, New Brunswick, Prince Edward Island, and Newfoundland and Labrador), the Territories (Yukon and Northwest Territories), and Canada. No public health Twitter accounts from the Canadian territory of Nunavut were identified. Additionally, we randomly selected tweets across the two broad account types (ie, agencies and decision makers). The resulting stratified random sample of 501 tweets about COVID-19 contained 58 tweets from Canadian national-level accounts, 52 from Alberta, 66 from British Columbia, 50 from the Maritimes, 199 from Ontario, 47 from the Prairies, 17 from Quebec, and 12 from territorial accounts. The sample of 501 tweets about COVID-19 also contained 377 tweets from agencies and 124 tweets from decision makers.

Before beginning the content analysis, 3 researchers (CS, CB, SS) were trained on a set of 50 tweets randomly selected from the overall sample of 6982 COVID-19 tweets. This was done so that the researchers could familiarize themselves with the various variables for coding and troubleshoot any issues with the definitions of the coded variables. To distribute the 501 tweets for the content analysis equally among the three researchers, the French-language tweets (n=27) were first identified and allocated to the researcher with fluency in French. Among the remaining English-language tweets, 50 were randomly selected and allocated to each of the 3 researchers so that these overlapping tweets could be used to calculate the Krippendorff α value for interrater reliability. This resulted in one researcher coding 201 tweets (including the 27 French tweets), and the other two coding 200 tweets each. To integrate the 50 tweets that all 3 researchers had coded into the overall sample, one researcher’s code was randomly selected from the 3 possible codes for each variable, such that the probability of selection was proportional to the frequency of that answer (eg, if two-thirds of coders agreed on a code, there was a 2 in 3 chance of that code being selected).

There were 10 coding variables in total. The first variable, media, captured the presence of media in the tweet and the type of media present (ie, image, video or document), if applicable. The next variable, message function, was coded using 3 mutually exclusive coding variables: information, action, or community. “Information” tweets included those whose main purpose was to inform, educate, or update the reader on case counts, disease transmission dynamics, policy changes, and COVID-19 symptoms. “Action” tweets included those whose main purpose was intended to prompt changes in the behaviors or actions of readers, such as encouraging social distancing, hygiene practices, or other harm-reducing behaviors. Finally, tweets were coded as “community” if their main purpose was community-building, identifying community supports and programs, or highlighting stories from or about the local community. Since threaded tweets could contain multiple message purposes, coding was based on the most prominent theme for the entire tweet thread. These variables for message function are consistent with those first proposed by Lovejoy and Saxton [40] for classifying the three main functions of organizations’ Twitter use and have been used in similar research [21,23,25].

The final variable, use of a risk communication strategy, was coded using 6 nonmutually exclusive coding variables: corrective, risk, efficacy, concern, uncertainty, and experts. Tweets were classified as “corrective” if they corrected some incorrect information about COVID-19 or aimed to prevent the spread of misinformation. Tweets were classified as “risk” if they contained information that would help a reader make a judgment about the risk of contracting COVID-19 or experiencing health complications from COVID-19. This included tweets containing information regarding absolute risks, relative risks, as well as the identification of high-risk subpopulations. Tweets were classified as “efficacy” if they referenced an individual’s or community’s ability to execute an action or activity successfully resulting in some tangible benefit to health or a reduction of harm related to COVID-19. Tweets were classified as “concern” if they acknowledged the fears, concerns, worry, or anxiety associated with COVID-19. Tweets were classified as “uncertainty” if they acknowledged uncertainty, confusion, or a lack of available information about COVID-19. Finally, tweets were classified as “experts” if they implicitly or explicitly mentioned some agreement, coordination or collaboration between public health experts or other credible health organizations or individuals. The presence of any one of these 6 variables was used to indicate the use of any risk communication strategy in the tweet and were based in part on best practices in communication identified by Seeger [31] used to improve organizational and individual responses during crisis events (see Multimedia Appendix 2 for dataset of 501 manually coded Tweets).

### Statistical Analysis

Each of the 3 coders worked independently through the same randomly selected 50 tweets, where each tweet had 10 variables to be codified. Krippendorff α [41] was used to measure the interrater agreement among coders, which was calculated using the R package *irr* [42]. Overall, interrater reliability was considered high (α=.829), with all 3 coders reporting total agreement on 453 out of the 500 answers (90.6%). Any codification of unstructured phenomena can have subjective biases, including when there is only one coder. However, the computed level of reliability suggests that there was largely internal agreement amongst the classification of variables within the sample such that results are less likely to be an artifact of internal disagreement or bias.

To assess whether differences in the mean number of retweets per tweet across each message function category were statistically significant, the nonparametric Kruskal-Wallis one-way ANOVA (analysis of variance) test was used since our data were not normally distributed.

We used the nonparametric Mann–Whitney–Wilcoxon Test to assess differences in the mean number of retweets per tweet between tweets containing at least one risk communication strategy and tweets containing no risk communication strategy.

## Results

### COVID-19 Tweets by Account Type

Our sample comprised 32,737 tweets, which included individual tweets and threads authored by 118 Canadian public health Twitter accounts (agencies and decision makers) between January 1, 2020, to June 30, 2020. Approximately 21% (n=6982) of all tweets contained content about COVID-19. Table 1 summarizes the characteristics of tweets in our sample by account type. Medical officers of health authored the largest percentage of tweets about COVID-19 relative to their total tweets (n=1337, 35%), representing the largest contribution of any account type. Conversely, accounts that belonged to provincial health ministers authored the smallest percentage of their tweets about COVID-19 (n=350, 18%). Accounts corresponding to Canadian medical officers of health also had the highest average number of retweets for COVID-19–related tweets, as well as the largest total follower count (summed across accounts in this category) at 416,611 total users (range 213–206,288 followers).

**Table 1:** Number of tweets and follower counts by account type, January 1, 2020, to June 30, 2020.

Account type	Twitter accounts, n	Total tweets^a^, n	Mean tweets per account	Tweets about COVID-19, n (%)	Mean retweets per tweet about COVID-19	Total follower count^b^, n (range)
Public health agencies	4	2272	568	524 (23)	60	407,546 (10,201-325,112)
Regional and local health departments	69	19,919	289	3832 (19)	10	406,108 (194-82,347)
Provincial health authorities	15	4778	319	939 (20)	13	170,387 (23-41,779)
Medical officers of health	22	3859	175	1337 (35)	112	416,611 (213-206,288)
Provincial health ministers	8	1909	239	350 (18)	52	134,019 (908-53,325)

^a^Equals the number of tweets and/or tweet threads authored by Twitter accounts per type between January 1 and June 30, 2020.

^b^Corresponds to the number of followers the accounts had at the end of the study period on June 30, 2020.

**Figure 1:**
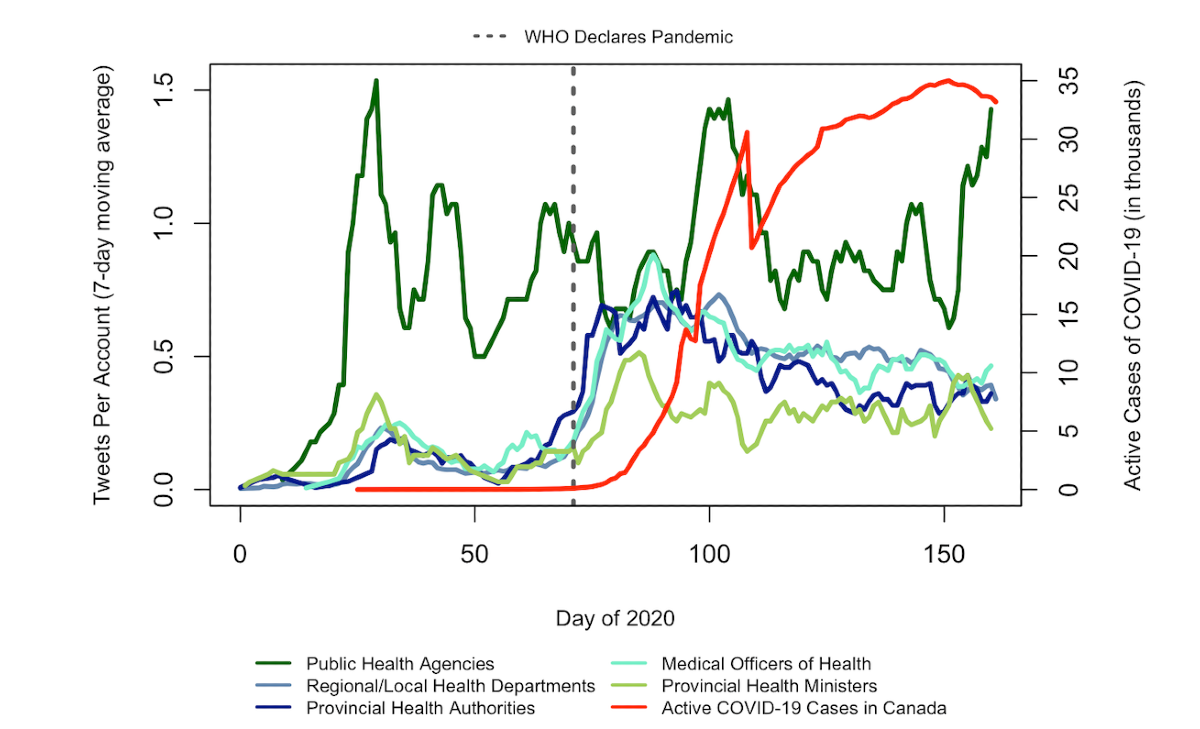
Daily rate of COVID-19 tweets by account type, January 1, 2020, to June 30, 2020. WHO: World Health Organization.

Figure 1 displays longitudinal trends in the daily rate of COVID-19 tweets stratified by account type using a 7-day moving average. All account types had an increase in their daily rate of COVID-19–related tweets after January 25 (day 25), when Canada’s first case of COVID-19 was reported (Figure 1). Compared to the other account types, public health agencies authored the highest number of COVID-19–related tweets per account through most of the period studied. An exception to this trend was observed shortly after the WHO declared COVID-19 a pandemic, when the other account types increased their frequency of tweets about the disease. In the time between the WHO’s declaration (day 70) and when Canada’s COVID-19 cases first peaked (day 108), the daily number of COVID-19 tweets per account for regional and local health departments, provincial health authorities, and medical health officers appeared to converge (Figure 1).

### Engagement

**Figure 2:**
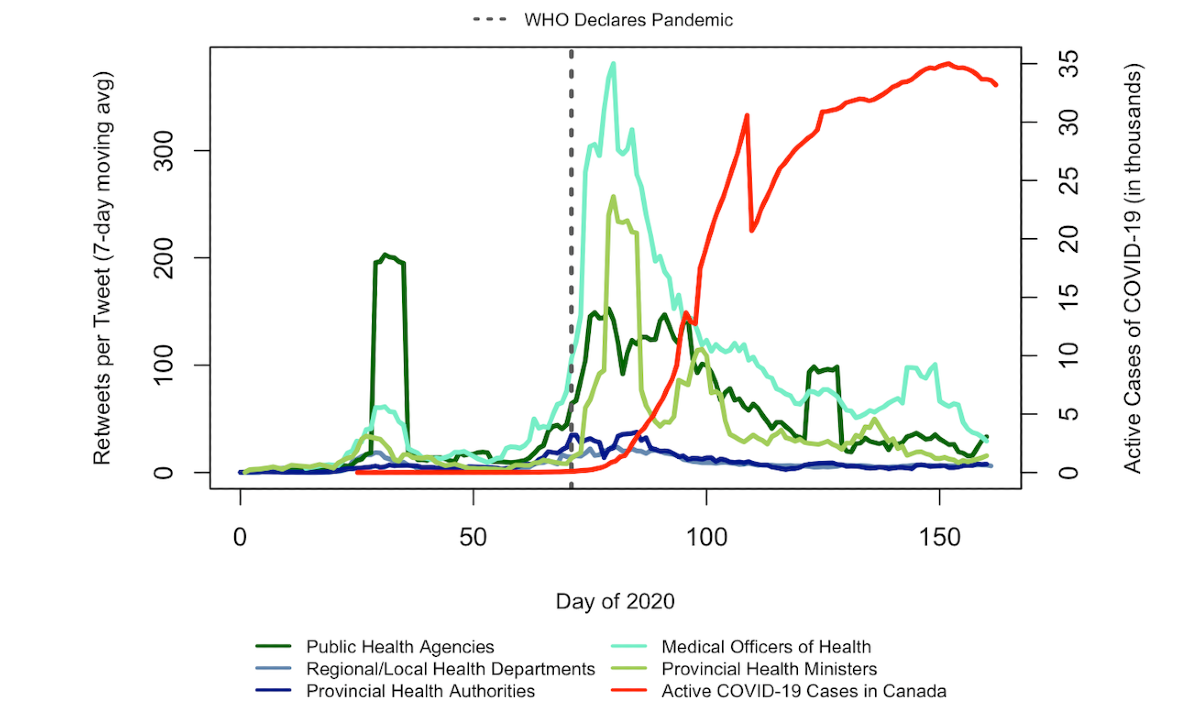
Daily retweets per COVID-19 tweet by account type, January 1, 2020, to June 30, 2020. Avg: average; WHO: World Health Organization.

Figure 2 displays trends in the average number of retweets for COVID-19–related tweets over time by account type. Around day 28, 3 days after the first case of COVID-19 was confirmed in Canada, there was a large spike in the average number of retweets for public health agencies that lasted a few days before returning to baseline. For all account types, the next period of increase in retweets per tweet occurred around the time of the WHO’s pandemic declaration (day 70). However, a few weeks after the pandemic was declared, retweets appeared to trend downward, even before COVID-19 cases peaked in Canada (Figure 2). The maximum daily average in retweets (381 retweets per tweet) was seen on day 80 among accounts belonging to medical officers of health; importantly, this was the day that Canada announced it would be closing its border to most noncitizens and nonpermanent residents. In contrast to other account types, medical officers of health maintained relatively high engagement (average of 50 or more retweets per COVID-19–related tweet) for a sustained period, beginning shortly before the WHO’s pandemic declaration on day 70 and lasting until the second peak in COVID-19 cases in Canada on day 150. For accounts corresponding to provincial health ministers, daily retweets peaked on the same day as they did for medical health officers (day 80) but trended downwards shortly thereafter (Figure 2). Trends in retweets over time were similar for provincial health authorities and regional or local health departments (Figure 2).

Table 2 summarizes the total number of tweets containing each engagement strategy, stratified by account type, as well as the sum and mean number of retweets by Twitter engagement strategy. User mentions were used less frequently than other engagement strategies and received the lowest mean retweets across all accounts. The most frequently used engagement strategies across all account types were hashtags (n=4798) and URLs (n=4781). These two engagement strategies appeared equally as frequently in tweets authored by public health agencies; however, the use of media (eg, images and videos) was associated with the highest average retweet count (67 retweets per tweet) for this account type. Similar findings were observed for provincial health authorities, which also used URLs most frequently (n=669) and received the highest average retweets on tweets containing media (18 retweets per tweet). Although accounts corresponding to regional and local health departments also incorporated URLs in more tweets than the other strategies (n=2766), they received the highest average retweets for tweets that contained hashtags (13 retweets per tweet). Tweets by regional and local health departments on average received fewer retweets per tweet compared to the other account types. Tweets by medical health officers that contained hashtags on average received the highest number of retweets per tweet compared to any engagement strategy and any account (134 retweets per tweet). Both medical officers of health and provincial health ministers used tweets containing media in fewer than half of their tweets, while the other three account types used media in more than half of their tweets.

**Table 2:** Summed tweet (n=6982) and retweet frequencies and percentages by engagement strategy and account type, January 1, 2020, to June 30, 2020.

Account type and engagement strategy		Number of tweets^a^ containing each engagement strategy, n (%)	Summed retweets for all tweets containing each strategy, n	Mean retweets per tweet containing each strategy
**Public health agencies**				
	Media	350 (67)	23,291	67
	Hashtags	451 (86)	29,418	65
	URLs	451 (86)	24,788	55
	User mentions	139 (27)	2654	19
**Regional and local health departments**				
	Media	2640 (69)	30,223	11
	Hashtags	2462 (64)	30,824	13
	URLs	2766 (72)	27,136	10
	User mentions	753 (20)	4648	6
**Provincial health authorities**				
	Media	477 (51)	8555	18
	Hashtags	614 (65)	8333	14
	URLs	669 (71)	6671	10
	User mentions	326 (35)	1644	5
**Medical officers of health**				
	Media	342 (26)	25,924	76
	Hashtags	1058 (79)	141,841	134
	URLs	681 (51)	61,697	91
	User mentions	396 (30)	15,098	38
**Provincial health ministers**				
	Media	157 (45)	5015	32
	Hashtags	213 (61)	14,261	67
	URLs	214 (61)	8134	38
	User mentions	135 (39)	3756	28

^a^Equals the number of tweets and/or tweet threads authored by Twitter accounts per type between January 1 and June 30, 2020.

### Content Analysis

During the content analysis of 501 tweets, 16 tweets were identified by the 3 researchers as not being directly related to content about COVID-19, resulting in a data set of 485 tweets about COVID-19. When coding the tweets for message function, 21 tweets were found to not have a classifiable purpose and were omitted from this part of the analysis. Table 3 summarizes the frequency and percentage of tweets identified as information, action, and community for each of the five account types in the sample. More than half of all coded tweets authored by public health agencies were classified as information tweets (n=17, 52%), which received the highest average number of retweets per tweet (56 retweets per tweet) compared to action (43 retweets per tweet) and community tweets (12 retweets per tweet) for these accounts. In our sample, tweets authored by regional and local health departments were most often classified as action tweets (n=101, 47%), and on average these received the highest number of retweets per tweet for this account type (10 retweets per tweet). Tweets corresponding to provincial health authorities, medical health officers, and provincial health ministers were mostly classified as information (n=47, 47%; n=56, 58%; and n=10, 53%, respectively); however, action tweets authored by each of these account types received more retweets per tweet on average compared to their information tweets (12, 259, and 44 retweets per tweet, respectively). The difference in mean retweets across the three message functions was not statistically significant (*P*=.18). However, when examining only those tweets that received one or more retweet (n=359), the difference between mean retweets across the three message functions was statistically significant (*P*<.001).

**Table 3:** Summed tweet (n=464) and retweet frequencies and percentages by message function and account type, January 1, 2020, to June 30, 2020.

Account type and message function		Number of tweets^a^ with each message function, n (%)	Summed retweets for all tweets of each function, n	Mean retweets per tweet of each function
**Public health agencies**				
	Information	17 (52)	957	56
	Action	13 (39)	554	43
	Community	3 (9)	36	12
**Regional and local health departments**				
	Information	51 (24)	325	6
	Action	101 (47)	1043	10
	Community	64 (30)	223	3
**Provincial health authorities**				
	Information	47 (47)	393	7
	Action	33 (33)	388	12
	Community	19 (19)	142	7
**Medical officers of health**				
	Information	56 (58)	6172	103
	Action	30 (31)	7765	259
	Community	11 (11)	223	20
**Provincial health ministers**				
	Information	10 (53)	293	27
	Action	5 (26)	221	44
	Community	4 (21)	90	23

^a^Equals the number of tweets and/or tweet threads authored by Twitter accounts per type between January 1 and June 30, 2020.

Table 4 summarizes the frequencies and percentages of risk communication strategies used by account type for the stratified random sample of COVID-19 tweets that were coded during the content analysis. Overall, the risk communication strategies that we examined were not very widely used and appeared only in 262 tweets out of our sample of 485 tweets. Since some tweets in our sample contained more than one type of risk communication strategy, as a result, there were 334 strategies used across the 262 tweets. Efficacy statements were the most commonly used strategy (efficacy accounted for more than one-third of the strategies used by each account type), and this strategy appeared in 163 of 262 tweets containing any risk communication strategy. For accounts corresponding to public health agencies, efficacy (n=12, 46%) and risk (n=8, 31%) statements were the most frequently used strategy; however, tweets containing these strategies were not the most retweeted. Instead, a single tweet thread containing corrective information that was authored by PHAC (which addressed misinformation on COVID-19) received the most retweets (134 retweets per tweet). Among regional and local health departments, the second most frequently used risk communication strategy after efficacy was addressing concern about COVID-19; however, their tweets containing corrective information received the most retweets on average (30 retweets per tweet). Provincial health authorities and medical health officers used risk statements at similar frequencies (n=12, 23% and n=24, 22%, respectively); however, tweets containing this strategy had fewer retweets on average among both account types when compared to tweets containing other risk communication strategies. Although medical health officers only authored 4 tweets containing statements that acknowledged the uncertainty around COVID-19, these tweets received the highest number of retweets per tweet compared to the other strategies used by that account type (358 retweets per tweet).

**Table 4:** Summed risk communication strategies (n=334) and percentages and retweet frequencies by strategy and account type, January 1, 2020, to June 30, 2020.

Account type and risk communication strategy^a^		Number of risk communication strategies^b^ used by account type, n (%)	Summed retweets for all tweets containing each risk communication strategy, n	Mean retweets per tweet containing each strategy
**Public health agencies**				
	Corrective	1 (4)	134	134
	Risk	8 (31)	112	14
	Efficacy	12 (46)	845	70
	Concern	2 (8)	154	77
	Uncertainty	1 (4)	67	67
	Experts	2 (8)	18	9
**Regional and local health departments**				
	Corrective	4 (3)	119	30
	Risk	5 (4)	20	4
	Efficacy	81 (60)	711	9
	Concern	24 (18)	236	10
	Uncertainty	10 (7)	90	9
	Experts	12 (9)	35	3
**Provincial health authorities**				
	Corrective	1 (2)	0	0
	Risk	12 (23)	38	3
	Efficacy	18 (34)	129	7
	Concern	10 (19)	220	22
	Uncertainty	3 (6)	5	2
	Experts	9 (17)	38	4
**Medical officers of health**				
	Corrective	4 (4)	767	192
	Risk	24 (22)	2709	113
	Efficacy	49 (45)	12,413	253
	Concern	13 (12)	3928	302
	Uncertainty	4 (4)	1433	358
	Experts	15 (14)	1695	113
**Provincial health ministers**				
	Corrective	0 (0)	N/A^c^	N/A
	Risk	2 (20)	123	62
	Efficacy	3 (30)	175	58
	Concern	1 (10)	43	43
	Uncertainty	0 (0)	N/A	N/A
	Experts	4 (40)	53	13

^a^Risk communication strategies were not mutually exclusive; therefore, a single tweet could contain multiple strategies.

^b^Equals the number of tweets and/or tweet threads authored by Twitter accounts per type between January 1 and June 30, 2020.

^c^N/A: not applicable.

**Figure 3:**
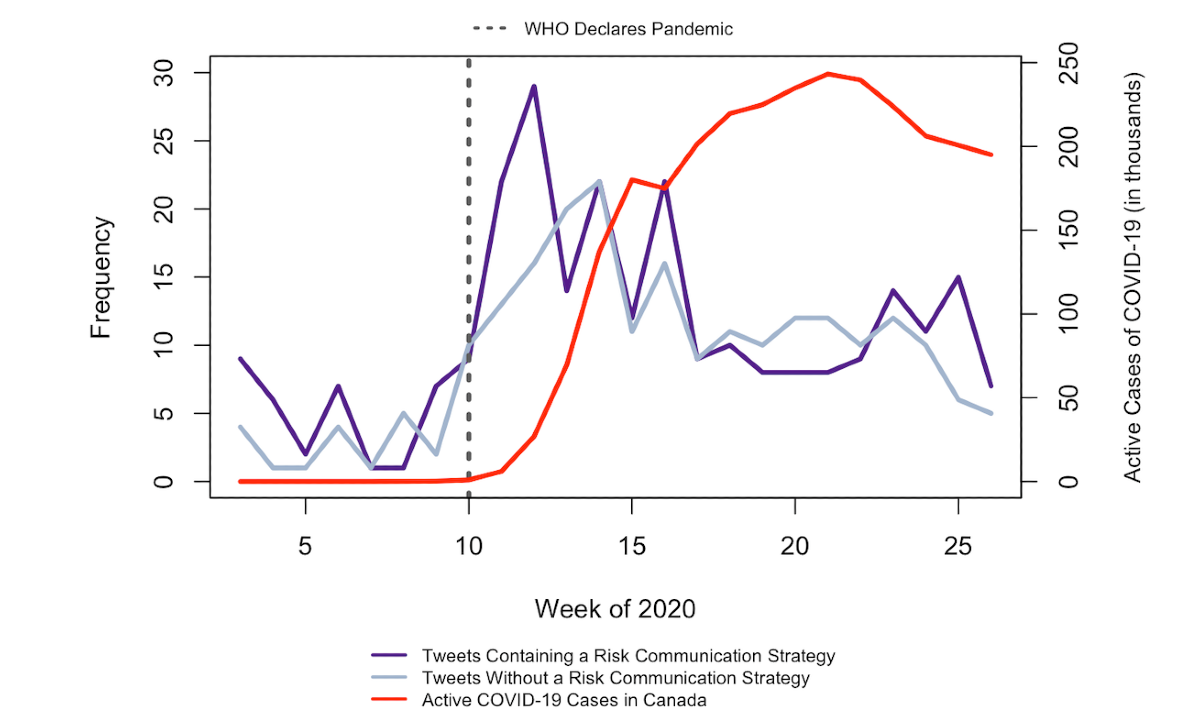
Weekly frequency of COVID-19 tweets containing any risk communication strategy, across all account types, January 1, 2020, to June 30, 2020. WHO: World Health Organization.

Figure 3 displays the weekly frequency of COVID-19 tweets containing any risk communication strategy and tweets containing no strategy across all account types for the stratified random sample of COVID-19 tweets that were coded during the content analysis. When examining trends across all account types, the use of risk communication strategies appeared to increase in tweets produced after the WHO’s pandemic declaration (week 10) but decreased in the period of time between the first and second peaks of COVID-19 cases in Canada (weeks 15 and 22, respectively). Tweets that did not contain a risk communication strategy were not tweeted as frequently as tweets that did contain a risk communication strategy shortly after the pandemic was declared. The use of any risk communication strategy by all account types appeared to increase again a little after the second peak of COVID-19 cases in Canada after week 22. Tweets using at least one risk communication strategy (n=262) received an average of 61 retweets per tweet, while tweets using no risk communication strategies received an average of 13. This nearly 5-fold difference was statistically significant (*P*<.001).

## Discussion

### Principal Results

#### RQ 1 and 2: Tweets and Retweets Over Time by Account Type

Our study of Canadian public health Twitter accounts revealed that tweeting practices and tweet engagement differed between various agencies and decision makers during the COVID-19 pandemic. Of the five account types that we examined, public health agencies and medical officers of health stood out for their tweeting frequency and the high engagement that their tweets received. These two account types had the largest percentage of tweets about COVID-19 relative to all their total number of tweets, as well as the highest daily rate of COVID-19 tweets. This finding is consistent with their roles as the primary agencies and individuals responsible for implementing and communicating a public health response during health crises in Canada. Our findings showed that these two account types also received the highest average engagement (retweets) during the study period, suggesting that Twitter users also recognize these agencies and individuals as the primary information sources to share with their peers during a public health crisis like COVID-19. These findings are positive in that if public health agencies and medical health officers continue to establish a consistent Twitter presence, attract followers, and engage their Twitter audiences, their communications may continue to be shared widely.

We observed that trends in the daily frequency of tweets by account type over time were generally consistent with changes in public concern and engagement over the course of the pandemic [27]. However, it is interesting to note that during key moments in time where the threats of COVID-19 were increasing, the daily rate of COVID-19 tweets did not always correspond to increased retweet counts, and instead trends in tweeting and retweeting varied by account type. For example, when the first case of COVID-19 was confirmed in Canada on January 25, 2020, public health agencies tweeted the most about COVID-19 and received the most retweets for these tweets compared to all other account types; however, after the WHO declared COVID-19 a pandemic on day 70, the daily rates of COVID-19 tweets for all account types began to increase and nearly converged. Medical officers of health received the most retweets per COVID-19 tweet during this period (day 80). These results suggest that Twitter users may have shifted their engagement from tweets authored by public health agencies to those by medical health officers; however, this trend was less evident by the time Canada’s COVID-19 cases peaked.

It is also interesting to note that daily rates of COVID-19 tweets authored by public health agencies seemed to peak around the same time that key moments related to COVID-19 took place in Canada (ie, after Canada’s first case, just before the first peak in COVID-19 cases, and shortly after the second peak in COVID-19 cases), which suggests that trends in case counts may have at least partially informed this account type’s tweeting practices. This trend was not seen for medical officers of health, whose daily rates of COVID-19 tweets trended downward after the WHO declared COVID-19 a pandemic, despite subsequent peaks in Canadian COVID-19 cases. This is important because increasing COVID-19 case counts partially inform the public about their risk of contracting COVID-19 and thus may lead to increased information seeking. As a result, more public health accounts should respond to increasing case numbers with increased daily tweeting about COVID-19, not less. Unfortunately, information seeking cannot be indicated by engagement metrics such as retweet counts; therefore, our results do not reflect which tweets were most seen or read by Twitter users. However, other research has noted that frequently updated social media feeds are perceived by audiences as more relevant during crises and should be updated enough so as to be noticed by users, regardless of whether they receive engagement [20].

Despite having lower daily COVID-19 tweet rates compared to public health agencies, medical officers of health and provincial health ministers received the highest daily retweets per tweet on day 80, 10 days after the WHO declared COVID-19 a pandemic. This finding was somewhat surprising given that past research has identified health agencies (especially at the federal level) as being able to garner more retweets than other public health accounts during past pandemics, largely due to their recognition and influence over online information sharing [33]. This finding may reflect a Twitter audience shift from governmental institutions to individuals, consistent with some research that has identified a spokesperson effect during public health crises, whereby people seek out a leading voice that is credible and relatable as their primary source of information [43].

One reason that medical officers of health may have been able to maintain a higher average retweet count for the remainder of the study period is that Twitter users may have perceived their content as more medically or scientifically relevant during the pandemic, which other studies have found can lead to more retweets [14,44], likely because expert accounts are perceived as highly authoritative and trustworthy information sources. Perhaps unsurprisingly, Twitter accounts corresponding to provincial health ministers had the lowest percentage of tweets about COVID-19, and the number of retweets that their tweets received decreased significantly after peaking on day 80. Given that provincial health ministers are elected public officials, often with little to no expertise in public health, our findings suggest that these individuals likely relegated responsibilities around COVID-19 communications to other accounts more focused on public health. These results are also consistent with prior research that found that the public may be more likely to distrust information from governments or elected officials and more likely to share information from sources perceived as more trusted or more “expert” (eg, physicians and medical researchers) [45].

#### RQ3: Use of Engagement Strategies

The frequent use of hashtags and URLs in the majority of COVID-19 tweets that we analyzed is consistent with other studies examining engagement to tweets authored by health agencies [22], and suggests that many Canadian public health agencies and decision makers are aware of and incorporate common Twitter engagement strategies in their tweeting practices. However, the use of these metrics was not always associated with higher engagement, suggesting that there is no single strategy to garner engagement. Hence, each account type may need to tailor their approach. Although user mentions were among the least used engagement metric, and received the lowest average retweets per tweet, the value of these types of tweets extends beyond engagement. In fact, user mentions are viewed as a way to establish dialogue between a tweeter and their audience, build relationships between users, and improve transparency and trust in tweeting institutions [46]. Other studies have also noted that organizations that do not fully utilize Twitter engagement strategies may be missing important opportunities to craft more interactive and engaging communications during a crisis [47].

#### RQ4: Tweet Message Functions and Use of Risk Communication Strategies

In our content analysis of a stratified random sample of tweets by Canadian public health agencies and decision makers, we found that of the three message functions that we examined, information tweets were most common across all account types, except regional and local public health departments, which used action tweets more frequently. This finding was consistent with other studies that have found tweets conveying information to be the most frequently tweeted by health organizations during other pandemics [26,48]; however, action tweets were most frequently produced in a study by Wahbeh et al [49] of physicians’ tweets on COVID-19. Despite information tweets being the most frequently used by the accounts in our sample, action tweets received on average more retweets per tweet for all account types except for public health agencies, which received on average more retweets for information tweets. Other research has also found that action tweets receive the most engagement compared to tweets with other message functions [21]. Our findings suggest that users may seek out and engage with different messages from different account types, relying more on public health agencies for information about COVID-19 and relying on the other accounts for instructions on actions and preventative measures they should be taking. During regular noncrisis periods, community tweets can help a public health agency build an online community and initiate a sense of togetherness [25]; however, the lack of engagement that these tweets received in our study suggest that the public’s need for information and direct actions during the COVID-19 pandemic may require public health agencies and decision makers to shift away from community-type tweets during a crisis to meet the needs of their audiences.

Furthermore, the results of our content analysis demonstrated that the risk communication strategies that we examined were not very widely used by any account type, appearing in just over half of the tweets that we analyzed. For example, our study found very few tweets provided corrective information that could be used to tackle misinformation about COVID-19, which is consistent with work by Guidry et al [28] that found only 1% of tweets by health organizations in their sample addressed misinformation on Ebola. These findings suggest that a lack of corrective tweets could represent a missed opportunity for public health agencies and officials to combat misinformation spread during a pandemic. It is also worth noting that risk tweets containing statements that would aid users in making a judgment about their risk of contracting COVID-19 or the harms associated with COVID-19 only appeared in approximately 11% of the tweets in our sample (n=51). In fact, only one category of the risk communication strategies that we examined (efficacy) appeared in at least one-third of the tweets authored by all account types, a frequency that was similar to the percentage of tweets containing efficacy statements about the Zika virus in a study of tweets authored by US public health agencies [33]. On the other hand, tweets that acknowledged concerns about COVID-19 tended to receive among the highest retweets per tweet in our study, which is consistent with risk communication literature identifying concern as an important strategy that aids the public in developing faith in communicators that demonstrate compassion [31].

Despite the overall lack of risk communication strategies employed in the tweets in our sample, our findings demonstrate that including one or more strategies was associated with more engagement on average compared to tweets that did not contain any risk communication strategies (61 retweets per tweet versus 13 retweets per tweet, respectively). We also found that risk communication strategies tended to be used at key moments during the COVID-19 pandemic. The use of risk communication strategies appeared to peak in the weeks just after the WHO declared COVID-19 a pandemic and trended slightly upward after each of the two peaks of COVID-19 cases in Canada. This finding is consistent with other studies that have found that risk communication becomes less prevalent over the course of a crisis [50], since this information is considered most valuable at the beginning of a crisis when uncertainty is high [35]. However, given that our understanding of COVID-19 transmission and health impacts is still developing, a lack of sustained risk communication in tweets by Canadian public health agencies and decision makers could be problematic if it leads to inaccurate perceptions of personal health risks or indifference toward public health measures.

With so much discussion of the pandemic online, supplying information users with high-quality, consistent messaging on the health risks associated with COVID-19 is critical for improving health literacy in the population [51]. Therefore, while the use of these risk communication strategies at key moments could be viewed as promising, more risk communication overall should be undertaken by all public health Twitter accounts to ensure that their audience continuously receives relevant, accurate, and up-to-date information on potential health risks related to COVID-19.

### Strengths, Limitations, and Future Work

One methodological advantage that sets our study apart from others is the use of tweet threads, in addition to individual tweets, as the unit of analysis, rather than analyzing each tweet from a thread on its own. Analyzing threads allowed for a more accurate examination of tweeting practices by recreating the message as it would have been viewed originally to a Twitter user. This was a major strength of our content analysis as it allowed for the entire message to be coded and analyzed, rather than a small segment of it. Since threads are commonly used to craft messages that would otherwise be impossible to fit within a tweet’s character limit, analyzing them individually would have provided an incomplete picture.

This research has several limitations related, in large part, to its reliance on Twitter data. First, we analyzed the tweets of public health agencies and decision makers in Canada who had tweeted between January 1 and June 30, 2020; however, there are numerous other authoritative health-related Twitter accounts and other official government accounts that tweeted about COVID-19 during this period (eg, physicians, health nongovernmental organizations, etc), which the public may have also engaged with. Moreover, not all members of the public use Twitter; therefore, engagement as measured by counts of retweets does not offer insights into which public health agencies or decision makers the broader Canadian public may consider most important. Additionally, our results may not reflect today or tomorrow’s Twitter landscape, and therefore only indicate tweeting practices at a snapshot in time. However, our study can offer a glimpse into trends on information sharing during the first 6 months of the COVID-19 pandemic based on what Canadian public health agencies and decision makers were tweeting and how Twitter users engaged with this content. Our study’s findings can be useful to those public health accounts that we included in our analysis as well as to other health organizations or individuals that may be looking for ways to better utilize Twitter to engage with users seeking health information on this platform.

The findings from our study could be improved through additional analysis of content authored by public health accounts on other social media platforms (eg, Instagram, Facebook, etc) or additional refinement of the categories we used for classifying the Twitter accounts and tweet content. For example, future work examining public health communications in Canada could build on our work by contrasting tweeting practices by province or other geographic elements, which could uncover more trends in information sharing and engagement given the region-specific administration of Canada’s public health policies. In addition, future work in this area could explore patterns in retweeted tweets and perform a network analysis to examine the Twitter interactions between various public health accounts.

### Conclusions

This study analyzed tweets by Canadian public health agencies and decision makers between January 1, 2020, and June 30, 2020, to examine their tweeting practices during the early stages of the pandemic. We also aimed to identify ways that tweets could be improved to effectively communicate risk and maximize engagement on this platform. Using a mixed methods approach, we conducted Twitter analytics and a qualitative content analysis to characterize the level of engagement that COVID-19 tweets authored by Canadian public health accounts received, as well as the purpose of their tweets and their use of key risk communication strategies. Our research findings suggest that while public health agencies authored more daily COVID-19–related tweets than other account types, engagement tended to be higher for tweets authored by medical health officers, particularly during key moments of the pandemic. Overall, most account types appeared to focus on disseminating information, with the exception of regional and local health departments, which tended to promote more action from users. Our results also point to the need for public health agencies and decision makers to monitor Twitter analytics in order to understand their audience and leverage whatever Twitter engagement strategies help maximize shares of their communications.

During the beginning of any crisis, the use of risk communication strategies by organizations and officials leading the response is critical to help inform the public about an often highly uncertain and rapidly evolving situation, address concerns, and instill trust in those leaders. Our study found that risk communication strategies were not widely used in tweets by any account type, even though these strategies were associated with more engagement. These findings suggest that Canadian public health agencies and decision makers may be missing an important opportunity to engage with information users about the mitigation of health risks related to COVID-19. Finally, our study builds on other literature that has explored differences in engagement to communications authored by individuals and institutions, and suggests that any medical officer of health or other expert individual currently not on Twitter should consider the platform as a means to disseminate information that Twitter users appear to be interested in sharing.

## Appendix

Multimedia Appendix 1 Flow chart of Tweet exclusion process.

Multimedia Appendix 2 Dataset of manually coded Tweets (n=501) from content analysis.

## Abbreviations

ANOVA: analysis of variance
PHAC: Public Health Agency of Canada
RQ: research question
SARS: severe acute respiratory syndrome
WHO: World Health Organization
