# Supplementary figures and images for: Examining Tweet Content and Engagement of Canadian Public Health Agencies and Decision Makers During COVID-19: Mixed Methods Analysis
Source: J Med Internet Res. 2021 Mar 11;23(3):e24883. doi: 10.2196/24883 (PMC7954113; doi:10.2196/24883)

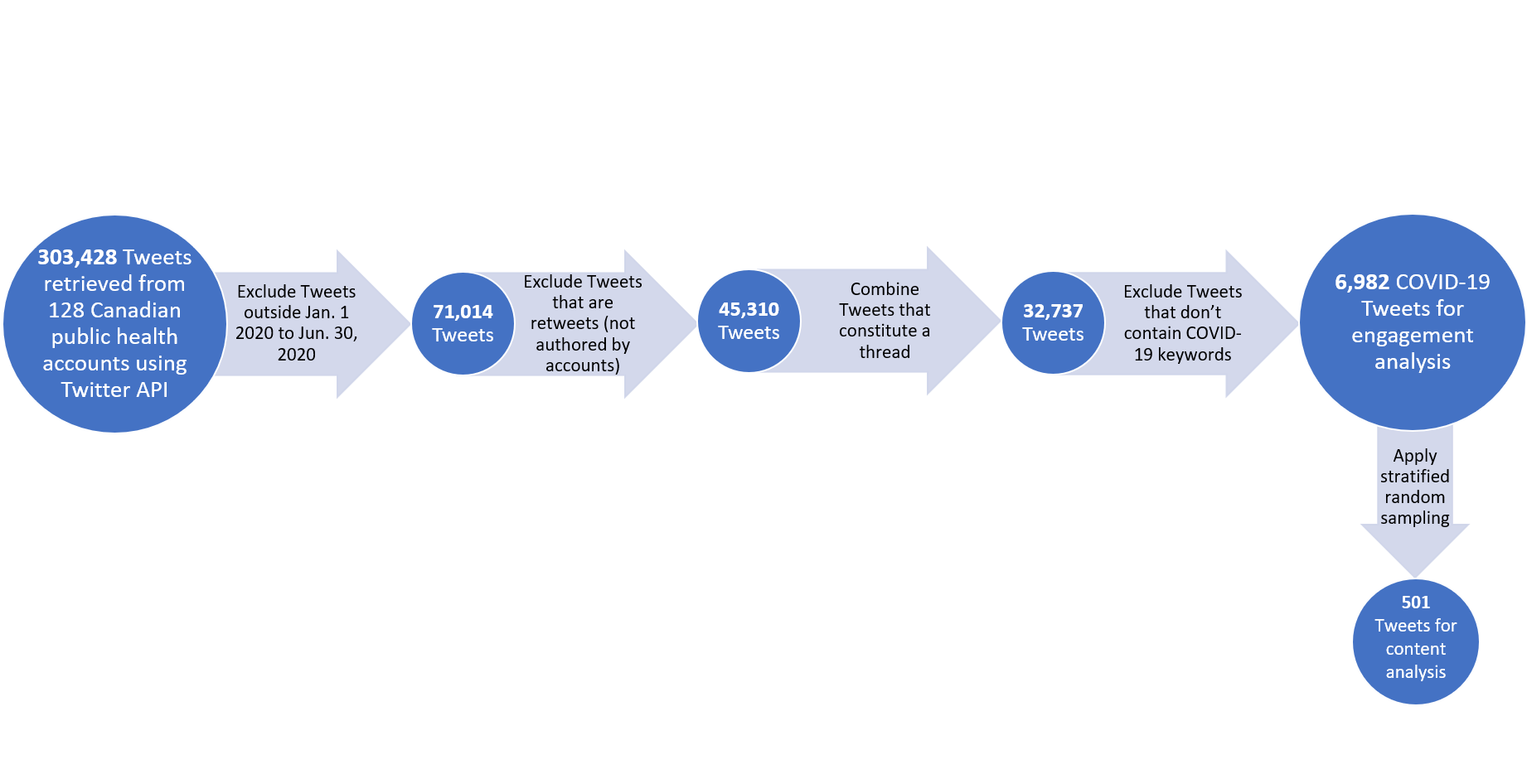

Supplement: Multimedia Appendix 1 [file jmir_v23i3e24883_app1.png]
